# Carmellose Mucoadhesive Oral Films Containing Vermiculite/Chlorhexidine Nanocomposites as Innovative Biomaterials for Treatment of Oral Infections

**DOI:** 10.1155/2015/580146

**Published:** 2015-04-30

**Authors:** Jan Gajdziok, Sylva Holešová, Jan Štembírek, Erich Pazdziora, Hana Landová, Petr Doležel, David Vetchý

**Affiliations:** ^1^Department of Pharmaceutics, University of Veterinary and Pharmaceutical Sciences, Palackého Třída 1-3, 612 42 Brno, Czech Republic; ^2^Nanotechnology Centre, VŠB-Technical University of Ostrava, 17 Listopadu 15/2172, Poruba, 708 33 Ostrava, Czech Republic; ^3^T4Innovations Centre of Excellence, VŠB-Technical University of Ostrava, 17 Listopadu 15/2172, Poruba, 708 00 Ostrava, Czech Republic; ^4^Department of Maxillofacial Surgery, University Hospital Ostrava, 17 Listopadu 1790/5, Poruba, 708 00 Ostrava, Czech Republic; ^5^Institute of Public Health Ostrava, Centre of Clinical Laboratories, Partyzánské Náměstí 7, 702 00 Ostrava, Czech Republic

## Abstract

Infectious stomatitis represents the most common oral cavity ailments. Current therapy is insufficiently effective because of the short residence time of topical liquid or semisolid medical formulations. An innovative application form based on bioadhesive polymers featuring prolonged residence time on the oral mucosa may be a solution to this challenge. This formulation consists of a mucoadhesive oral film with incorporated nanocomposite biomaterial that is able to release the drug directly at the target area. This study describes the unique approach of preparing mucoadhesive oral films from carmellose with incorporating a nanotechnologically modified clay mineral intercalated with chlorhexidine. The multivariate data analysis was employed to evaluate the influence of the formulation and process variables on the properties of the medical preparation. This evaluation was complemented by testing the antimicrobial and antimycotic activity of prepared films with the aim of finding the most suitable composition for clinical application. Generally, the best results were obtained with sample containing 20 mg of chlorhexidine diacetate carried by vermiculite, with carmellose in the form of nonwoven textile in its structure. In addition to its promising physicomechanical, chemical, and mucoadhesive properties, the formulation inhibited the growth of *Staphylococcus* and *Candida*; the effect was prolonged for tens of hours.

## 1. Introduction

The oral microflora is a very specific component of the human organism. It consists of aerobic and anaerobic microorganisms whose representation depends on several factors (e.g., age, composition of food, medication, lesions in the oral cavity, systemic diseases, or infections) [1, 2]. The most common ailment affecting the oral cavity is infectious stomatitis, which is caused by factors including inadequate oral hygiene, long-term use of antibiotics, smoking, presence of dental prostheses, and immunodeficiency or systemic disease (e.g., HIV, diabetes mellitus, and oncological illnesses), which allow the overgrowth of microorganisms and subsequent outbreak of inflammation in the oral cavity. Clinical manifestations during infectious stomatitis can be very annoying for the patient and can include pain, burning in the mouth, increased salivation, taste disturbances, and reduced food intake. The treatment of these ailments is usually based on the local application of various mucosal antiseptics in the form of rinses (e.g., chlorhexidine, cetylpyridinium, and triclosan) [3, 4] or systemic therapy (antibiotics and antimycotics) after the clinical status deteriorates [5–7].

This study deals with two representatives among the wide spectrum of microorganisms that have been identified as factors that can cause infectious stomatitis:* Candida albicans* and* Staphylococcus aureus*. Fungal infections of the oral mucosa are almost exclusively represented by oral candidiasis, of which* C. albicans* is the most common infectious agent [1, 2, 8]. Depending on the severity of the disease, treatment consists of the local or systemic administration of antiseptics and azole antifungal agents, improvement of oral hygiene, and good hygiene of any infected removable dental prostheses [9, 10].* S. aureus* is another problematic pathogen. It represents one of the most common causes of nosocomial infections that manifest in the oral cavity, especially in cases in which the mucosal barriers have been breached. After 1-2 days of incubation, serous exudation occurs in the injured tissue area and the surrounding oral mucosa becomes erythematous. If local treatment is unsuccessful, sepsis is a relatively common complication [11–13]. When treating infectious stomatitis caused by either* C. albicans* or* S. aureus*, the key factor is a successful local therapy capable of dealing with the disease before it spreads into the organism. As far as the treatment of oral infections is concerned, the current market lacks any type of local, long-acting application that can enable effective therapy without systemic treatment.

A possible solution might be the application of mucoadhesive oral films (MOFs) with incorporated inorganic clay molecules that act as carriers of antibacterial or antimycotic agents, which in turn are released gradually and directly at the target area in the oral cavity. This innovative medical formulation might satisfy the conditions of application comfort and might be especially suited to provide a long-term local effect, which would improve the effectiveness of the therapy and decrease its total strain on the body.

Mucoadhesion is a specific phenomenon of creating bonds during close contact between the mucoadhesive material and a biological surface covered with a mucus layer. Modern drug formulations based on this process have recently come to the foreground of therapeutic interest [14–21]. Thin, flexible films prepared by any of the proven methods (e.g., solvent casting, hot-melt extrusion, printing, or impregnation) are promising candidates for the oral administration of many drugs in order to ensure their systemic effect or local action in the oral cavity. Owing to their advantages (prolonged residence time, providing long periods of therapeutic drug levels at disease sites, and good stability of active ingredients), mucoadhesive films or patches represent the most recently developed medical formulation for oral application. Films are generally single- or multilayered laminates, which are preferred over adhesive tablets because they are flexible and comfortable to use. Because the film is thin and nonirritating and the structural polymers are strongly mucoadhesive, only minimal changes in the patients' normal activities (e.g., eating, drinking, or speaking) are necessary. Flexible patches of various sizes can be adapted to the morphology of the oral cavity and the size of the defect [15, 18].

Oral films with mucoadhesive properties represent a suitable matrix for incorporating a variety of drugs, either in their common form or bound to a specific carrier. Clay minerals appear to be very promising candidates that can act as excipients or as carriers of antimicrobial drugs [22, 23]. Clay minerals are hydrated aluminium phyllosilicates with a layered structure [24, 25]. Moreover, they are naturally occurring inorganic cation exchangers, and so they may undergo ion exchange with functional molecules and/or particles through an intercalation process, particularly with basic drugs. The layered clay mineral vermiculite (Ver) becomes a promising carrier in the area of antibacterial or antimycotic compounds because its layer charge is greater than that of the most commonly used montmorillonite (Mt). Antimicrobial compounds supported on a clay mineral matrix are generally known as inorganic and/or organic antibacterial materials. Inorganic cations of heavy metals (e.g., Ag^+^ [26–28], Cu^2+^ [29, 30], and Zn^2+^ [31]) are the ones used most often. Although clay-based inorganic materials show high thermal stability, they also have disadvantages, such as the accumulation of harmful heavy metals, mostly in the pseudohexagonal cavities of the silicate layers, resulting in decreased antibacterial activity. In spite of their low thermal stability, the organoclay materials exhibit many advantages compared to inorganic materials, mainly regarding their organophilicity, which results in easy adherence and ability to exterminate a number of bacterial species [32–35]. The antibacterial behaviour of vermiculite-bound chlorhexidine against selected Gram-positive and Gram-negative bacterial strains has already been demonstrated [36, 37].

To date, a wide variety of mucoadhesive materials have been used for the development of MOFs. Mucoadhesive polymers should exhibit certain physicochemical characteristics, including hydrophilicity, viscoelastic properties, and flexibility for interpenetration with mucus and epithelial tissue based on their numerous hydrogen bond-forming groups (hydroxylic, carboxylic, sulphate, or amide) [38–41]. The most used mucoadhesive polymers belong to the group of cellulose derivatives (e.g., hydroxypropyl methylcellulose, oxycellulose), acrylic derivatives, alginates, chitosan, polyoxyethylene, polyvinyl alcohol, thiolated polymers (thiomers), or materials that are able to adhere directly to the cell surface rather than to mucus [16], such as lectins or bacterial adhesives [14, 42–45].

Carboxymethyl cellulose (CMC), a derivative with carboxymethyl groups (-CH2-COOH) bound to some of the hydroxyl groups of the glucopyranose cellulose monomers, is among the most important mucoadhesive materials from the group of cellulose derivatives. This cellulose ether is obtained by carboxymethylating cellulose with sodium chloroacetate in an alkaline environment under strictly controlled conditions [46, 47]. CMC is commercially available in many forms that differ in their degree of substitution, viscosity, particle size, molecular weight, degree of polymerization, and other parameters [48]. The degree of substitution affects a number of physicochemical parameters of CMC, including viscosity, solubility, water-absorption capacity, and biological stability, among others [49]. CMC is a nonirritating and nontoxic material suitable for both external and internal use. It is also physiologically inert and partially biodegradable. Because of the mentioned advantages, it is widely used in medicine and in the pharmaceutical industry for a variety of functions, including to enhance wet wound healing and to act as a laxative, gelling agent, emulsion stabilizer, thickener, binder (in solid formulations), or a carrier of polymer for the formulation of ocular inserts. The mucoadhesive properties of CMC have also recently come to the fore because they are used in the oral, ocular, nasal, pulmonary, and vaginal application of modern formulations that feature controlled drug release [50–54].

Despite the intense focus on buccal film-based systems, there are no standardized methods for evaluation of their physicomechanical and chemical properties (e.g., residence time, mucoadhesive strength, and mechanical durability). This lack of standardized evaluation methods limits the possibility of comparing obtained data and evaluating the significance of formulation and process variables on the properties of the resulting films [21]. It is often difficult or impossible to use simple statistical methods to obtain information about the influence of variables or their combinations on the properties of final MOFs. It is appropriate to make simplifications that allow the expression of a large number of variables with a smaller number of so-called latent variables to determine dependency (correlation) in a multidimensional data set. Latent variables (principal components) then represent a kind of dimension in which the effect of variables is expressed collectively. Their advantage is that they are independent (orthogonal), which greatly simplifies interpretation. Principal component analysis (PCA) is one of the oldest and most widely used multivariate methods and is used primarily for exploratory data analysis [55].

This paper follows the previously published results aimed at the evaluation of toxicological and antibacterial properties of vermiculite nanocomposites [56]. The presented study describes the unique approach of using the solvent casting or impregnation methods to prepare MOFs from carmellose (a well-established mucoadhesive polymer) with the incorporation of a nanotechnologically modified clay mineral (vermiculite) and intercalated antiseptic drugs (chlorhexidine diacetate and digluconate). We used multivariate data analysis methods to evaluate the influence of the formulation and process variables on the physicomechanical and chemical properties of MOFs. This evaluation was complemented by testing the antimicrobial and antimycotic activity of MOFs, with the aim of finding the best composition for possible clinical application.

## 2. Materials and Methods

### 2.1. Materials

Clay mineral vermiculite (Mg^2+^Ver) from Letovice (Czech Republic) was used for the experiment. Mg^2+^Ver obtained from a weathered zone of the ultrabasic body of metamorphosed basalts in the Letovice complex, in the eastern part of the Bohemia Massif (Czech Republic), was milled in a planetary mill and sieved, and the fraction <45 *μ*m was used for the experiment. This sample did not contain other mineral phases identifiable by X-ray diffraction. Its crystallochemical formula, calculated from the results of the elemental chemical analysis, was (Si_3.13_Al_0.86_Ti_0.02_)(Mg_2.53_Fe_0.45_Al_0.02_)O_10_(OH)_2_(Mg_0.19_K_0.01_Ca_0.02_) per O_10_(OH)_2 _with a cation exchange capacity (CEC) of 140 cmol(+)/kg. Chlorhexidine diacetate (abbreviated CA, C_22_H_30_N_10_Cl_2_·2C_2_H_4_O_2_, Sigma Aldrich) and chlorhexidine digluconate (abbreviated CG, C_22_H_30_N_10_Cl_2_·2C_6_H_12_O_7_, 20% in H_2_O, Sigma Aldrich) were employed as active ingredients to prepare organovermiculite nanocomposites, and ethanol was used as a solvent.

Carmellose sodium was employed (NaCMC, Blanose type 7LF-PH, Ashland Aqualon Functional Ingredients, USA), as a semisynthetic cellulose derivative, as the basic mucoadhesive and film-forming structural polymer in the formulation of MOFs. In some samples (Table 1), an acid form of carmellose (HCMC; Hcel HT, Holzbecher Medical, CZ) was incorporated into the structure of the film as a nonwoven textile to improve its physicomechanical and chemical properties. In all cases, glycerol (Gly) (Kulich, CZ) acted as a plasticizer at a concentration of 3%. Mucin from porcine stomach (Type II, Sigma-Aldrich, Co., USA) as a 5% dispersion (w/w) in phosphate buffer pH 6.8 according to European Pharmacopoeia [57] was used to prepare artificial mucus for the testing of mucoadhesive properties. All other chemicals used in this experiment were of analytical grade.

### 2.2. Designation of the Samples

The number in the sample name expresses the amount of the active ingredient in one MOF (10 versus 20 mg); CHDAC means abbreviation of used chlorhexidine diacetate, CHDAG of chlorhexidine digluconate; C is the control (no active ingredient) and T after dash means if there was nonwoven textile or not.

### 2.3. Preparation of Organovermiculites

The solutions of CA and CG were prepared in ethanol in concentrations according to the 0.5 × CEC of Mg^2+^Ver and then stirred and heated with Mg^2+^Ver suspended in water. After centrifugation, solid products were dried, and samples for the experiment were named Mg^2+^Ver_CA and Mg^2+^Ver_CG.

### 2.4. Preparation of Mucoadhesive Films

A four percent dispersion of NaCMC was prepared by swelling the polymer in distilled water for 24 h and subsequently stirring using Ultra-turrax (T 25 basic, IKA, WERKE, GmbH&Co.KG, D) for 2 min (16,000 rpm). As a plasticizer, glycerol was added to a final concentration of 3% (w/w) by continual mixing. Mg^2+^Ver_CA and Mg^2+^Ver_CG were added to the casting dispersion at two different concentrations (Table 1) to ensure that the amount of chlorhexidine in final MOFs was 10 or 20 mg, which corresponds to the dosage applied during rinsing of the oral cavity with commercially available mouthwashes. Subsequently, ten different batches of MOFs were prepared, five using the solvent casting method (samples without HCMC textile) and five using the innovative method of impregnation of the textile from acid form of carmellose (Table 1).

#### 2.4.1. Solvent Casting Method

Using an automatic pipette (Transferpette S, range 5 to 20 mL, Brand, UK), 18 mL of the prepared uniform dispersions was cast into a round plastic mold (63 mm diameter), and the solvent was left to evaporate for 24 h in a ventilated oven at 30°C. Samples (25 × 25 mm, 10 × 40 mm, and 15 mm diameter) of final films for testing of physicomechanical and chemical properties and* in vitro* antimicrobial activity were punched using steel punches.

#### 2.4.2. Method of Impregnation

The acid form of carmellose in the form of nonwoven textile was cut into a circular shape (63 mm diameter) and placed in the casting molds. The textile was impregnated with the same amount of prepared dispersions to ensure the same concentration of active ingredients as in samples prepared using the solvent casting method. Solvent was evaporated at 30°C for 24 h in a ventilated oven, and prepared samples were the same sizes as were used for the solvent casting method.

### 2.5. Characterization of Vermiculite Samples

X-ray diffraction (XRD) patterns of organovermiculite samples were recorded using the RIGAKU Ultima IV diffractometer (reflection mode, Bragg-Brentano arrangement, CuK*α* radiation) in ambient atmosphere under constant conditions (2–60° 2*θ*, scan speed 2°/min, 40 kV, 40 mA). The IR spectra of organovermiculite samples were obtained using the KBr method with a NEXUS 470 Fourier transform (FTIR) spectrometer (Thermo Nicolet, USA). The spectrometer was equipped with a Globar IR source, KBr beam splitter, and DTGS detector. For each spectrum, 128 scans were obtained with resolution of 4 cm^−1^. Range of measurements was 400–4000 cm^−1^. The particle size (PS) of Mg^2+^Ver and organovermiculite samples was determined using laser diffraction particle size analyzer HORIBA LA-950 with two short-wavelength blue and red light sources in conjunction with forward and back-scatter detection to enhance sizing performance in the range 0.01–3000 *μ*m.

The morphology of initial Mg^2+^Ver and prepared organovermiculite samples was investigated by scanning electron microscope (SEM) Philips XL 30. SEM images were obtained using back-scatter detector (BSE) and accelerating voltage 20 kV.

### 2.6. Physicomechanical, Chemical, and Morphological Properties of MOFs

The weight of MOFs was measured on ten circular (15 mm diameter) samples selected at random from each batch, which were individually weighed on the analytical balance (KERN 870 - 13, Gottl. KERN & Sohn GmbH, D). The results were expressed as the average weight of the film and its standard deviation.

Film thickness was evaluated by microscopic analysis using an optical microscope (STM-902 ZOOM, Opting, CZ) and colour digital camera (DFW X700, Sony, JPN). The rectangular sample of the film was vertically fixed in a holder; the microscope was focused on the edge of the film, and the sample thickness was measured at five different places on the film. This process was repeated three times per sample type.

Surface pH was measured using a contact pH meter (Flatrode, Hamilton, CH). A moistened pH meter electrode was dipped into the MOF, and the value was recorded after stabilization (approximately 60 s). The measurement was repeated three times per sample.

**Figure 1:**
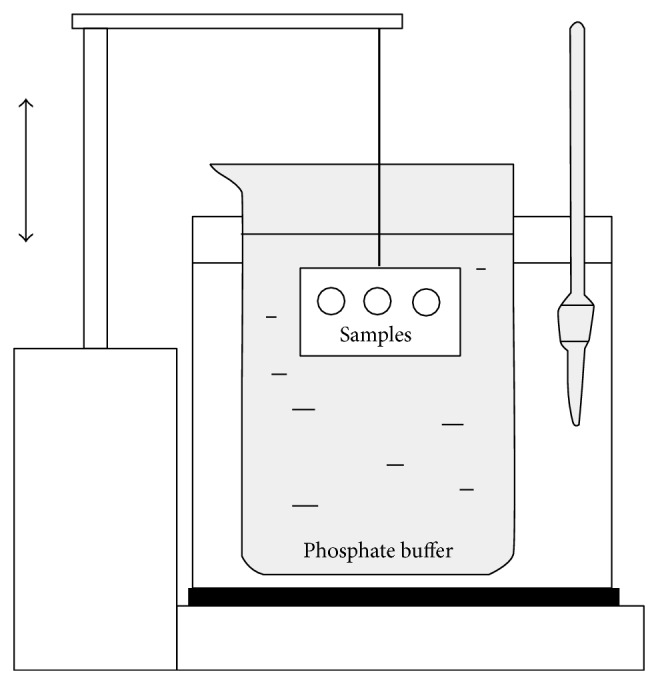
Modified disintegration apparatus for determination of* in vitro *residence time.

A modified disintegration apparatus was used to determine* in vitro *residence time according to Nafee et al. [58] (Figure 1). A standard basket for tablet insertion was replaced with a plastic slab that was vertically fixed to the apparatus. Oral mucosa was simulated using a cellophane membrane glued to the surface of the slab and covered with a 5% mucin dispersion (w/w) in phosphate buffer (pH 6.8; 10 *μ*L/cm^2^). The phosphate buffer (pH 6.8) [56] was maintained at 37°C and used as testing medium. The slab with attached circular MOF samples (15 mm diameter) was allowed to move up and down (samples were completely immersed in the buffer solution at the lowest point and were out of the solution at the highest point.) The time necessary for complete detachment or erosion of the film from the surface was recorded (Table 2). This measurement was repeated three times per sample.

A modification of Shidhaye's method was used to evaluate the mechanical properties of the prepared films [59]. A CT3 Texture Analyzer (Brookfield, USA) equipped with a 4.5-kg load cell was used for tensile testing of the prepared MOFs. Film samples (10 × 40 mm) were held between two clamps of probe TA-DGA positioned at a distance of 2 cm. The lower clamp was held stationary, and the upper clamp moved at a rate of 0.5 mm/s to pull apart the strips of mucoadhesive layers until the strip broke. The strength and work done during this process and the deformation of the film (elongation) at the moment of tearing were measured. This measurement was repeated three times per sample.

The texture analyzer with a TA39 cylindrical probe (2 mm diameter) was used for a puncture test. The strength needed to puncture square samples (25 × 25 mm) fixed in the JIG TA-CJ, the work done during this process, and the deformation of the film at the moment of penetration were measured. The layer with sedimented drug or textile material was oriented downwards, and the mucoadhesive layer faced upward. This measurement was repeated three times per sample.

Because the films were of different thicknesses and were prepared using different methods (solvent casting or impregnation), values measured by the texture analyzer were recalculated for the film thickness 100 *μ*m for better comparison (Table 3).

As well as in the case of solid samples, the morphology of prepared MOFs was characterized by SEM Philips XL 30. SEM images were obtained using back-scatter detector (BSE) and accelerating voltage 20 kV.

### 2.7. Multivariate Data Analysis

Methods of principal component analysis (PCA) were used for descriptive evaluation of the experimental data. Prior to modeling, the variables were adjusted by autoscaling, that is, mean centering and scaling by standard deviation. The influence of formulation variables (Table 1) on the parameters of mechanical resistance,* in vitro* residence time, and surface pH was subsequently evaluated using multiple linear regression (MLR) with use of analysis of variance (ANOVA). MLR models was assessed on the basis of characteristics such as *R*-square regression (which describes each model's explained variability), *R*-square of prediction (which expresses the model's predictive ability), and coefficient of variation (CV%; the average modeling error expressed as a percentage of the mean). Statistical evaluations were conducted using the program Unscrambler X (v. 10.3, Camo Software, NOR).

### 2.8. Antimicrobial Tests

#### 2.8.1. Organovermiculites

The minimum inhibitory concentration (MIC) of the prepared organovermiculite samples was defined as the lowest concentration that completely inhibited bacterial growth. Dilution and cultivation were performed on 96-well microtitration plates. The highest applied concentration of active substance was 10% (w/v). The dispersions were further diluted using a threefold dilution method in glucose stock in such a manner that the second through the seventh rows of wells contained sample dispersed in concentrations of 3.33%, 1.11%, 0.37%, 0.12%, 0.04%, and 0.01%. The eighth row of wells contained pure glucose stock as a control test. Glucose suspensions of* S. aureus* CCM 3953 (1.1 × 10^9^ CFU/mL) and* C. albicans *ATC90028 (1.1 × 10^9^ CFU/mL), 1 *μ*L each, provided by the Czech collection of microorganisms were applied to the wells. One microliter of each microorganism suspension was transferred (after 30, 60, 90, 120, 180, 240, and 300 min, and then at 24-h intervals for 5 days) from each well into 100 *μ*L of fresh glucose stock and incubated at 37°C for 24 and 48 h. Antibacterial activity was evaluated by turbidity, which indicates bacterial growth [60]; that is, lower turbidity correlates with greater growth inhibition.

#### 2.8.2. Mucoadhesive Oral Films

The suspension of* S. aureus* (50 *μ*L, density 10^8^/mL) and/or* C. albicans* (50 *μ*L, density 10^8^/mL) was applied to MOFs with a surface area of 1.77 cm^2^ (circular sample, 15 mm diameter). Both strains were incubated under the same conditions (dark, 37°C, Petri dish). Subsequently, MOFs were investigated by direct imprinting on solid culture medium (blood agar for* S. aureus*;* Sabouraud agar* for* C. albicans*) at time periods of 30, 60, 120, 180, 240, and 300 min and then at 24, 48, 72, and 96 h. After incubation (24 and 48 h), the growth of bacteria and yeast strains was observed and expressed as the number of colony forming units (CFU) on the surface of the imprint [60].

## 3. Results and Discussion

### 3.1. Characterization of Vermiculite Samples

**Figure 2:**
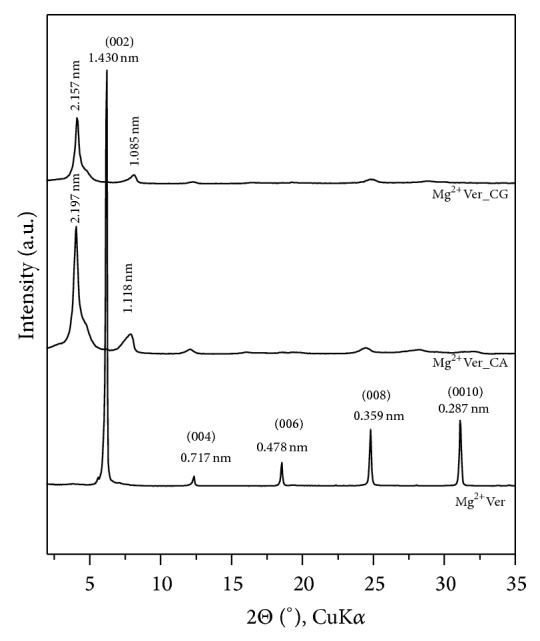
XRD patterns of natural Mg^2+^Ver and the organovermiculite samples Mg^2+^Ver_CA and Mg^2+^Ver_CG.

The XRD pattern of the natural Mg^2+^Ver showed the sequence of the basal reflections (Figure 2). The value *d*(002) = 1.430 nm confirmed the presence of two layers of water molecules around the exchangeable cations in the interlayer space [61, 62]. Treatment of Mg^2+^Ver with CA and/or CG at concentrations of 0.5 × CEC led to intercalation, which expanded the space between the layers and resulted in the appearance of a new reflection, designated on the XRD patterns as* d* = 2.197 nm (Mg^2+^Ver_CA) and* d* = 2.157 nm (Mg^2+^Ver_CG). Another new reflection in the XRD patterns of both organovermiculites of approximately 1 nm corresponded to the dehydrated vermiculite phase [62, 63].

**Figure 3:**
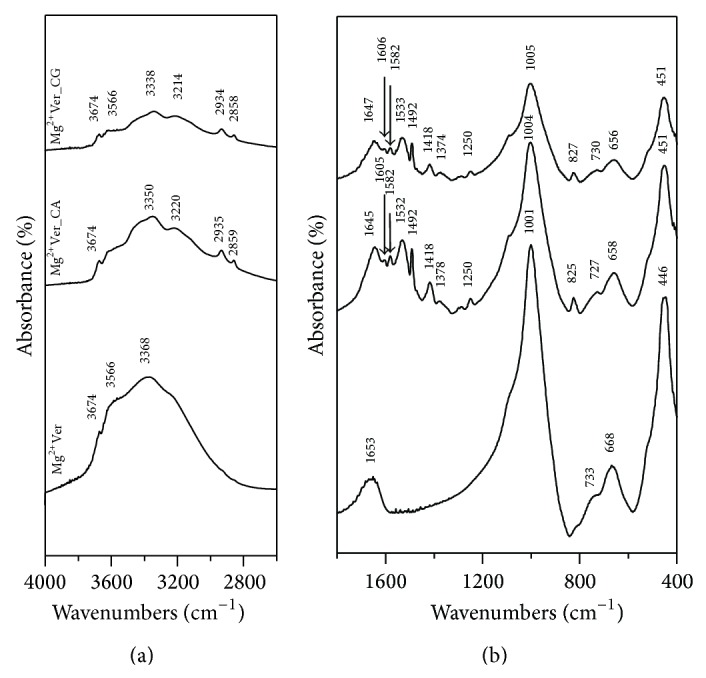
IR spectra of natural Mg^2+^Ver and the organoclay samples Mg^2+^Ver_CA and Mg^2+^Ver_CG.

The IR spectrum of Mg^2+^Ver (Figure 3) shows a band at 3674 cm^−1^ in the OH stretching region attributed to the Mg_3_OH unit; and absorption at 668 cm^−1^ belonging to the OH bending vibration. These bands suggest that vermiculite has a trioctahedral character [64]. Absorption at 3566 cm^−1^ in the OH stretching region belongs to the Fe_2_OH unit. The presence of this band indicates that although vermiculite is nominally trioctahedral, some of the OH groups are associated with vacancies and are in a locally dioctahedral environment [64]. The absorption observed at 3368 cm^−1^ corresponds to the OH stretching vibration of adsorbed water, and the adsorption observed at 1653 cm^−1^corresponds to the OH bending vibration of adsorbed water. Finally, an intensive band at 1001 cm^−1^ was assigned to the Si-O stretching vibration together with the Si-O bending vibration at 446 cm^−1^ [65]. The IR spectra of organovermiculites Mg^2+^Ver_CA and Mg^2+^Ver_CG (Figure 3) showed new bands at 3350 and 3338 cm^−1^, which correspond to the asymmetric NH stretching bands, and those at 3220 and 3214 cm^−1^ correspond to the symmetric NH stretching bands of CA and CG, respectively [66, 67]. Bands at 2935, 2934 and 2859, 2858 cm^−1^ are assigned to asymmetric and symmetric C-H stretching bands of CA and CG, respectively [66, 67]. The bands found in the 1580–1490 cm^−1^ region in both organovermiculite spectra originate in the NH bending vibration of secondary amine and imine groups [66, 67]. Because the C=N stretching vibration of the imine group appears near 1645 cm^−1^, it is difficult to distinguish because this band overlaps the water bending vibration of Mg^2+^Ver. The absorption at 1418 cm^−1^ belongs to the C=C stretching vibrations of the aromatic ring and the C-H out-of-plane deformation vibrations of the 1,4-disubstituted aromatic ring near 825 cm^−1^.

The particle size (PS) parameters were measured by the laser diffraction method in liquid mode. The parameters obtained from measurements are median diameter (*d*_50_) and the volume-weighted mean diameter (*d*_43_). Table 2 shows growth of these parameters after treatment of natural Mg^2+^Ver with antimicrobial drugs.

**Figure 4:**
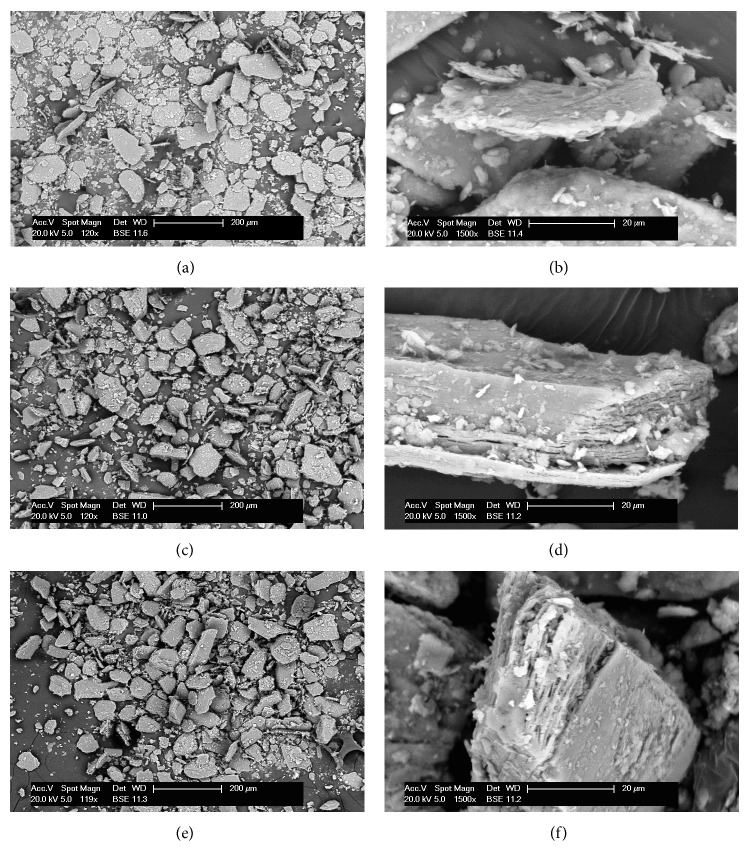
SEM photographs of parent clay and organoclay samples. (a) Mg^2+^Ver (120x); (b) Mg^2+^Ver (1500x); (c) Mg^2+^Ver_CA (120x); (d) Mg^2+^Ver_CA (1500x); (e) Mg^2+^Ver_CG (120x); (f) Mg^2+^Ver_CG (1500x).

SEM images of natural clay Mg^2+^Ver and organovermiculite samples are shown in Figure 4, magnified 120x and 1500x. The particle size is not obvious from the images in 120x magnification (Figures 4(a), 4(c), and 4(d)). For this reason, particle size distribution analysis was preferred. On the other hand from images of organovermiculite samples in 1500x magnification (Figures 4(d) and 4(f)) the enlargement of clay layers could be seen due to the intercalation of antimicrobial drugs into natural clay Mg^2+^Ver structure (Figure 4(b)). These results correspond with the results form XRD analysis, in which antimicrobial drugs were intercalated into interlayer space of clay.

### 3.2. Physicomechanical, Chemical, and Morphological Properties of MOFs

Overall, the weight of the prepared films ranged from values of 64.67 ± 3.97 mg (sample C) to 144.96 ± 10.08 mg (sample 20CHDG-T) (Table 3), which are not problematic for oral application. In general, MOFs with nonwoven textile exhibited greater weights than samples prepared by solvent casting method. The addition of an organoclay composite to the samples also increased their weight. Generally, films without active agent showed the lowest weight (C: 64.67 ± 3.97 mg; CT: 75.89 ± 8.80 mg) (Table 3). In contrast, films with 20 mg of intercalated chlorhexidine diacetate and chlorhexidine digluconate weighed the most (20CHDAC: 130.60 ± 5.21 mg; 20CHDAC-T: 140.80 ± 6.16 mg; 20CHDG: 131.42 ± 8, 29 mg; 20CHDG-T: 144.96 ± 10.08) (Table 3). Films containing chlorhexidine digluconate composite were of slightly greater weights than films containing chlorhexidine diacetate, although they contained the same amount of the active agent and textile material (Table 3).

The thickness of the prepared MOFs ranged from 256.25 ± 12.26 *μ*m (C) to 679.53 ± 25.34 *μ*m (20CHDG-T) (Table 3). MOFs without drugs were the thinnest (C: 256.25 ± 12.26 *μ*m; CT: 344.36 ± 17.75 *μ*m) (Table 3). Films with 20 mg of chlorhexidine diacetate or chlorhexidine digluconate had the greatest average thickness (20CHDAC: 522.53 ± 12.29 *μ*m; 20CHDAC-T: 662.09 ± 26.27 *μ*m; 20CHDG: 522.11 ± 15.69 *μ*m; 20CHDG-T: 679.53 ± 25.34 *μ*m) (Table 3). With the same amount of active agent and same textile material present, thicker MOFs were produced from organoclay containing chlorhexidine digluconate than chlorhexidine diacetate (Table 3). Films without textile samples containing 20 mg drug were of almost the same thickness (20CHDAC: 522.53 ± 12.29 *μ*m; 20CHDG: 522.11 ± 15.69 *μ*m), but films with 10 mg chlorhexidine diacetate were thicker than films with 10 mg chlorhexidine digluconate (10CHDAC: 383.12 ± 1.96 *μ*m; 10CHDG: 372.21 ± 4.23 *μ*m). The optimal thickness of buccal film with adequate mechanical durability and mucoadhesive properties and without interference in the oral cavity ranged from 50 to 1000 *μ*m; all of the MOF sample films were within this range [68, 69].

pH value of the mucoadhesive films is also one of the important factors of their quality. Normal saliva pH is between 5.6 and 7.0. If the pH of the applied mucoadhesive dosage formulation is too acidic or too alkaline, it could cause local irritation in the oral cavity. Irritation of the buccal mucosa may lead to increased salivation, resulting in excessive hydration of the mucoadhesive films, faster dissolution of the film-forming polymers, and faster erosion of the mucoadhesive bonds. The result is usually an insufficient residence time on the buccal mucosa [16, 41, 70].

Films with incorporated nonwoven textile from an acid formulation of CMC have been assumed to exhibit lower pH values (Table 3). This observation results from the pH of the aqueous extract of the textile material, which ranges from 3.5 to 5.0 [71]. The addition and increasing amount of organoclays with both forms of chlorhexidine to the formulation increased the pH of the samples (Table 3). This phenomenon might be explained by the basic nature of chlorhexidine [72]. It was also observed (Table 3) that films containing chlorhexidine diacetate exhibited higher pH values and films with chlorhexidine digluconate exhibited lower pH values (while maintaining the same amount of active ingredient and the same representation of nonwoven textile). This might be explained by the p*K*_a_ of the acids forming the chlorhexidine salts. Lower p*K*_a_ value implies that the given acid is of a stronger nature. The p*K*_a_ of gluconic acid is 3.70, while the p*K*_a_ of acetic acid is 4.74. Gluconic acid is therefore the strongest of these two acids, and films containing chlorhexidine digluconate exhibited lower pH values.

The* in vitro* residence time was evaluated in order to determine the mucoadhesion ability of MOFs, as well as the influence of the presence of organoclay and nonwoven textile on this characteristic [69, 73]. The presence of nonwoven textile and the addition of chlorhexidine organoclay composite each increased the* in vitro* residence time of the mucoadhesive film on the artificial mucosa (Table 3).

Films containing nonwoven textile from the acid form of carmellose remained on the artificial buccal mucosa longer than samples without the textile (from 25.58 ± 5.31 min (C-T sample) to 75.03 ± 11.43 min (20CHDAC-T sample)). This observation might be explained by their greater mechanical strength and the strong ability of carmellose to bind to biological surfaces [18]. The effect of chlorhexidine organoclay might be explained by the substantivity phenomenon of chlorhexidine, which could be described as its ability to bind to buccal mucosal structures [74, 75].

The results of the MLR model for films with incorporated active substance (*R*-square > 0.70; *R*-square prediction > 0.50; CV% < 13, model *P* < 0.01) indicated that the nonwoven textile had a statistically significant effect on the* in vitro* residence time (*P* < 0.001). It was disabled to confirm the statistical significance of the addition of different type of chlorhexidine on the residence time of MOFs, because of high levels of noise in the measurements.

Texture analysis was employed to evaluate the mechanical properties of the prepared MOFs. Films were characterized in two different ways: tensile testing (tensile strength, tensile work, and elongation) and puncture testing (puncture strength, puncture work, and deformation). The results of these measurements are summarized in Table 4.

It was observed that the incorporation of the nonwoven textile into the structure of MOFs influenced the properties of prepared films. Films with incorporated nonwoven textile exhibited higher strength and less elongation than films without the textile (Table 4). The measured elongation values ranged from 14.65 mm (20CHDG) to 28.27 mm (C) for films without textile and from 3.20 mm (20CHDAC-T) to 5.30 mm (C-T) for films with textile (Table 4). The strength required to break the sample was 4.21 ± 0.52 N (20CHDG) to 9.49 ± 0.23 N (C) for films without textile and 5.60 ± 0.21 N (20CHDG-T) to 9.81 ± 0.16 N (C-T) for films prepared using the impregnation method (Table 4). The resulting tensile work was lower for samples without textile than for samples with textile (Table 4). The measured values of tensile strength and elongation indicated that the samples without the nonwoven textile were softer and more flexible, while the samples with textile were less flexible and possessed greater stiffness. Adding chlorhexidine organoclay reduced the elongation values (Table 4).

In samples without nonwoven textile, the deformation of films during puncture testing varied from 2.72 mm (20CHDAC) to 4.72 mm (C). In contrast, films with incorporated nonwoven textile exhibited deformation in the range of 1.76 mm (20CHDAC-T) to 2.79 mm (C-T) (Table 3). The strength required to puncture the films without nonwoven textile was 2.38 ± 0.09 N (20CHDG) to 6.01 ± 0.43 N (C), versus 2.75 ± 0.22 N (20CHDG-T) to 6.83 ± 0.18 N (C-T) for films prepared by impregnation (Table 4). Samples without nonwoven textile exhibited greater degrees of deformation, and less strength was needed to puncture them (Table 4). The resulting work required to puncture the sample, which is related to both the strength needed and the deformation rate, was generally less for samples without textile than for samples with nonwoven textile in their structure (Table 4). Films without textile were more flexible and exhibited greater softness. Conversely, films with nonwoven textile deformed less under pressure, and greater strength and more work were generally required to puncture them. This observation indicated the reduced flexibility and greater hardness/durability of these samples. The addition of chlorhexidine organoclay reduced the strength needed to puncture the samples, as well as their deformation. This effect became stronger as the amount of active material in the MOFs increased (Table 4).

**Figure 5:**
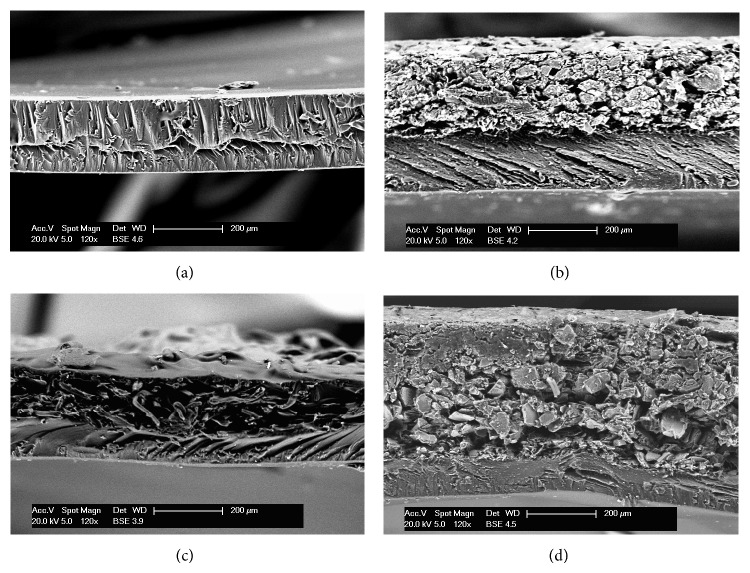
SEM photographs (120x) of MOFs structure. Samples: (a) C; (b) 20CHDAC; (c) C-T; (d) 20CHDAC-T.

The morphology of the prepared MOFs was evaluated by SEM (all the presented photographs are made in the 120x magnification). From the SEM images of pure carmellose film, C (Figure 5(a)), and MOF with antimicrobial nanocomposite, 20CHDAC (Figure 5(b)), it is evident that organoclay sample was anchored on the mucoadhesive polymer surface (Figure 5(b) upper layer is organoclay Mg^2+^Ver_CA and bottom layer is carmellose).  Figure 5(c) shows carmellose film with nonwoven textile (C-T), where nonwoven textile creates a rough surface (upper side of film). Image Figure 5(d) belongs to carmellose film with nonwoven textile and anchored antimicrobial nanocomposite (20CHDAC-T). In this case, it is evident that layer of organoclay Mg^2+^Ver_CA was anchored on carmellose film.

**Figure 6:**
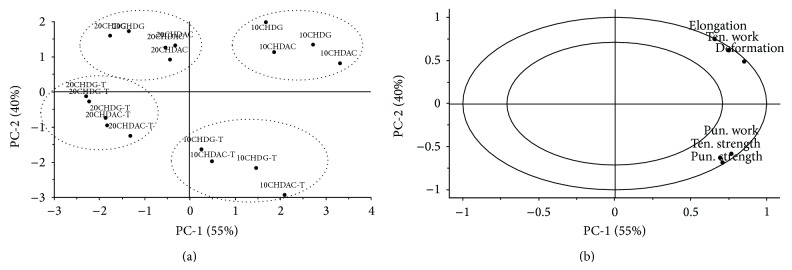
Principal component analysis. (a) Scores plot. (b) Correlation loadings plot.

The chosen properties of films prepared with active substances were also evaluated using PCA and MLR. The objects were distributed into four groups in the space of the first two components based on the presence of nonwoven textile and concentration of active substance (Figure 6(a)). Principal components are explained by variables shown in the PCA correlation loadings plot (Figure 6(b)), where variables near each other are strongly correlated.

MLR modeling was performed with the aim of obtaining models that were able to determine the effects of formulation variables from films prepared with the active substance on different mechanical properties (*R*-square > 0.80; *R*-square of prediction > 0.65; CV% < 20; models *P* < 0.001). MLR confirmed that the presence of nonwoven textile had significant negative effects on elongation, deformation, and tensile work (*P* < 0.001 for all) and significant positive effects on tensile strength, puncture work, and puncture strength (*P* < 0.01 for all). This trend is also apparent in the PCA correlation loadings plot (Figure 6(b)), in which the described variables are correlated in the opposite PC-2 space based on presence of the textile (Figure 6(a)). In this experiment, nonwoven textile increased the strength of films prepared from a 4% dispersion of NaCMC; that result was in contrast to the previous experiment, in which the addition of a nonwoven textile reduced the strength of films prepared from a 2% dispersion of NaCMC [21]. Furthermore, the concentration of active substance had a significant negative effect on all observed textural variables (*P* < 0.001), which could be illustrated by their correlation in the right part of the PC-1 in the correlation loadings plot (Figure 3(b)) based on the concentration of active substance (Figure 6(a)).

### 3.3. Antimicrobial Tests

#### 3.3.1. Organovermiculites

Antimicrobial tests were performed against the bacterial strain* S. aureus* (STAU) and the yeast* C. albicans* (CAAL). The activity of prepared organoclays was observed at various time periods.

The MIC values of Mg^2+^Ver_CA and Mg^2+^Ver_CG (Table 5) indicated that both samples were effective against STAU after ≥24 h. The MIC values for this microorganism were determined for both Mg^2+^Ver_CA and Mg^2+^Ver_CG samples at a concentration of 0.01% (w/v). Slightly worse results were obtained with both organovermiculite samples against CAAL after ≥24 h (MIC was 0.37% for Mg^2+^Ver_CA, and 1.11% for Mg^2+^Ver_CG). However, these samples gave better results against yeast in short time intervals (from 120 min), in contrast to STAU (Table 5).

#### 3.3.2. Mucoadhesive Oral Films

Antimicrobial tests against the bacterial strain* S. aureus* and the yeast* C. albicans* were performed with mucoadhesive films by direct imprinting on a solid culture medium at various time periods. Tables 6 and 7 depict the colony forming units (CFU) of prepared mucoadhesive films and control samples C and C-M. From all of the prepared MOFs, samples 10CHDAC-T, 20CHDAC-T, 10CHDG-T, and 20CHDG-T were the most effective against STAU (complete inhibition of STAU growth in maximum 1.5 h) (Table 6). The least effective samples against STAU were 10CHDAC and 10CHDG, which inhibited growth after >48 h (Table 6). The most effective MOFs against CAAL were 10CHDAC-T and 20CHDAC-T (Table 7). These samples were also able to inhibit yeast growth after >24 h. It could be concluded that all of the prepared samples exhibited good effectiveness regarding STAU growth inhibition and that samples with nonwoven carmellose textile incorporated into their structure exhibited better results. This might be because of the acidic condition of the nonwoven textile, which was also observed with respect to the surface pH of prepared MOFs (Table 3). The effectiveness of MOFs against CAAL was not satisfactory for all prepared samples. This observation was closely connected with which model drug was used; chlorhexidines are more effective against bacteria than against microorganisms (yeasts).

## 4. Conclusion

This study was aimed to prepare, test, and statistically evaluate mucoadhesive oral films based on the prospective mucoadhesive polymer carmellose in the form of its sodium salt and acid nonwoven textile. Films were formulated using two promising techniques: solvent casting and impregnation. Innovative nanotechnologically modified clay mineral (vermiculite) with intercalated antiseptic drugs, chlorhexidine diacetate and digluconate, was incorporated into their structure. Multivariate data analysis was used to evaluate the effects of the nonwoven textile and incorporation of the active substance on the physicomechanical, chemical, and mucoadhesive properties of formulated MOFs. These evaluations were complemented by testing of the antimicrobial and antimycotic activity of MOFs, which demonstrated the suitability of the prepared formulation for clinical use.

## Figures and Tables

**Table 1:** Composition of casting dispersions (% w/w) (g).

Sample	NaCMC	Gly	Mg^2+^Ver_CA	Mg^2+^Ver_CG	Water	HCMC textile
C	4	3	—	—	ad 100	No
C-T	4	3	—	—	ad 100	Yes
10CHDAC	4	3	11.17	—	ad 100	No
10CHDAC-T	4	3	11.17	—	ad 100	Yes
20CHDAC	4	3	22.34	—	ad 100	No
20CHDAC-T	4	3	22.34		ad 100	Yes
10CHDG	4	3	—	11.17	ad 100	No
10CHDG-T	4	3	—	11.17	ad 100	Yes
20CHDG	4	3	—	22.34	ad 100	No
20CHDG-T	4	3	—	22.34	ad 100	Yes

18 mL of casting dispersion was used for preparing of films with diameter of 63 mm.

**Table 2:** Particle size analysis.

Sample	*d*_50_ [*μ*m]	*d*_43_ [*μ*m]
Mg^2+^Ver	28.85	37.43
Mg^2+^Ver_CA	44.93	49.47
Mg^2+^Ver_CG	74.15	82.11

**Table 3:** Physical properties of prepared mucoadhesive oral films.

Sample	Weight (mg)	Thickness (*µ*m)	Surface pH	Residence time (min)
C	64.67 ± 3.97	256.25 ± 12.26	6.58 ± 0.01	25.58 ± 5.31
C-T	75.89 ± 8.80	344.36 ± 17.75	4.74 ± 0.01	82.33 ± 4.87
10CHDAC	95.11 ± 8.11	383.12 ± 1.96	7.66 ± 0.07	69.44 ± 2.63
10CHDAC-T	107.97 ± 6.11	436.39 ± 19.33	5.32 ± 0.07	96.46 ± 3.60
20CHDAC	130.60 ± 5.21	522.53 ± 12.29	7.94 ± 0.04	75.03 ± 11.43
20CHDAC-T	140.80 ± 6.16	662.09 ± 26.27	6.43 ± 0.07	84.95 ± 12.77
10CHDG	96.43 ± 10.68	372.21 ± 4.23	7.46 ± 0.07	58.83 ± 13.29
10CHDG-T	110.01 ± 5.12	474.32 ± 22.56	4.86 ± 0.11	84.11 ± 6.81
20CHDG	131.42 ± 8.29	522.11 ± 15.69	7.76 ± 0.10	60.01 ± 11.82
20CHDG-T	144.96 ± 10.08	679.53 ± 25.34	5.11 ± 0.05	97.36 ± 9.54

**Table 4:** Mechanical properties of prepared mucoadhesive oral films.

Sample	Tensile testing			Puncture testing		
	Tensile strength (N)	Tensile work (mJ)	Elongation (mm)	Puncture strength (N)	Puncture work (mJ)	Deformation (mm)
C	24.31 ± 0.60	325.00 ± 30.62	28.27 ± 3.49	15.40 ± 1.11	29.02 ± 2.90	4.92 ± 0.17
C-T	33.79 ± 0.57	176.70 ± 1.67	5.30 ± 0.05	23.52 ± 0.62	28.64 ± 1.15	2.79 ± 0.12
10CHDAC	27.63 ± 3.31	317.71 ± 39.35	19.19 ± 1.52	15.29 ± 0.96	22.58 ± 1.36	3.60 ± 0.04
10CHDAC-T	40.14 ± 1.42	156.28 ± 15.31	4.66 ± 0.04	22.65 ± 1.14	28.44 ± 1.58	2.20 ± 0.12
20CHDAC	32.34 ± 2.25	332.64 ± 34.28	15.47 ± 0.79	14.34 ± 0.64	19.96 ± 0.91	2.72 ± 0.04
20CHDAC-T	42.39 ± 1.23	116.14 ± 13.59	3.20 ± 0.02	21.08 ± 0.59	33.42 ± 0.65	1.76 ± 0.03
10CHDG	24.40 ± 1.61	299.96 ± 10.18	21.39 ± 0.37	13.98 ± 1.70	22.35 ± 3.20	3.65 ± 0.23
10CHDG-T	38.09 ± 0.37	167.28 ± 31.11	4.80 ± 0.23	23.29 ± 1.07	28.84 ± 2.55	2.45 ± 0.02
20CHDG	21.98 ± 2.72	227.15 ± 46.96	14.65 ± 2.27	12.41 ± 0.48	18.88 ± 1.22	2.77 ± 0.11
20CHDG-T	38.06 ± 1.44	136.70 ± 20.64	3.97 ± 0.51	18.67 ± 1.52	28.76 ± 1.42	1.89 ± 0.01

**Table 5:** Minimum inhibitory concentration (MIC) values (% w/v) of Mg^2+^Ver_CA and Mg^2+^Ver_CG.

Bacteria/yeast	Sample	MIC (% w/v)											
	Exposition	30 min	60 min	90 min	120 min	180 min	240 min	300 min	1 day	2 days	3 days	4 days	5 days
STAU	Mg^2+^Ver_CA	10	3.33	10	3.33	10	0.37	0.37	0.01	0.01	0.01	0.01	0.01
	Mg^2+^Ver_CG	10	10	10	10	3.33	3.33	3.33	0.01	0.01	0.01	0.01	0.01

CAAL	Mg^2+^Ver_CA	10	10	3.33	0.37	0.12	0.37	0.37	0.37	0.37	0.37	0.37	0.37
	Mg^2+^Ver_CG	10	10	10	3.33	1.11	1.11	1.11	1.11	1.11	1.11	1.11	1.11

**Table 6:** Antimicrobial properties of mucoadhesive oral films against *Staphylococcus aureus*.

Sample	CFU										
Exposition	30 min	60 min	90 min	120 min	180 min	240 min	300 min	1 day	2 days	3 days	4 days
10CHDAC	CN	CN	CN	CN	CN	CN	CN	55	**0**	0	0
10CHDAC-T	CN	**0**	0	0	0	0	0	0	0	0	0
20CHDAC	CN	CN	CN	CN	CN	CN	CN	**0**	0	0	0
20CHDAC-T	CN	**0**	0	0	0	0	0	0	0	0	0
10CHDG	CN	CN	CN	CN	CN	CN	CN	CN	**0**	0	0
10CHDG-T	**0**	0	0	0	0	0	0	0	0	0	0
20CHDG	CN	CN	CN	CN	CN	CN	CN	**0**	0	0	0
20CHDG-T	CN	7	**0**	0	0	0	0	0	0	0	0

C	CN	CN	CN	CN	CN	CN	CN	CN	CN	CN	CN
C-T	CN	CN	CN	CN	CN	CN	CN	CN	CN	CN	CN

Growth on glass	CN	CN	CN	CN	CN	CN	CN	CN	CN	CN	CN
Growth on blood agar	CN	CN	CN	CN	CN	CN	CN	CN	CN	CN	CN

CN (countless number: >300 CFU on the plate).

The initial time periods, in which microorganism growth was completely inhibited, are written in **bold** for each sample.

**Table 7:** Antimicrobial properties of mucoadhesive oral films against *Candida albicans*.

Sample	CFU										
Exposition	30 min	60 min	90 min	120 min	180 min	240 min	300 min	1 day	2 days	3 days	4 days
10CHDAC	CN	CN	CN	CN	14	11	10	11	**0**	0	0
10CHDAC-T	CN	CN	CN	CN	4	3	**0**	0	0	0	0
20CHDAC	CN	CN	CN	CN	CN	CN	26	29	0	0	0
20CHDAC-T	3	1	**0**	6	3	2	5	0	0	0	0
10CHDG	CN	CN	CN	CN	CN	CN	CN	CN	23	**0**	0
10CHDG-T	CN	CN	CN	CN	CN	CN	CN	CN	10	**0**	0
20CHDG	CN	CN	CN	CN	CN	CN	CN	100	**0**	0	0
20CHDG-T	CN	CN	CN	CN	CN	CN	15	13	8	**0**	0

C	CN	CN	CN	CN	CN	CN	CN	CN	CN	CN	CN
C-T	CN	CN	CN	CN	CN	CN	CN	CN	CN	CN	CN

growth on glass	CN	CN	CN	CN	CN	CN	CN	CN	CN	CN	CN
growth on blood agar	CN	CN	CN	CN	CN	CN	CN	CN	CN	CN	CN

CN (countless number: >300 CFU on the plate).

The initial time periods, in which microorganism growth was completely inhibited, are written in **bold** for each sample.
